# Measuring Public Concern About COVID-19 in Japanese Internet Users Through Search Queries: Infodemiological Study

**DOI:** 10.2196/29865

**Published:** 2021-07-20

**Authors:** Zhiwei Gao, Sumio Fujita, Nobuyuki Shimizu, Kongmeng Liew, Taichi Murayama, Shuntaro Yada, Shoko Wakamiya, Eiji Aramaki

**Affiliations:** 1 Social Computing Laboratory Graduate School of Science and Technology Nara Institute of Science and Technology Ikoma Japan; 2 Yahoo Japan Corporation Tokyo Japan

**Keywords:** COVID-19, search query, infodemiology, quantitative analysis, concern, rural, urban, Internet, information-seeking behavior, attitude, Japan

## Abstract

**Background:**

COVID-19 has disrupted lives and livelihoods and caused widespread panic worldwide. Emerging reports suggest that people living in rural areas in some countries are more susceptible to COVID-19. However, there is a lack of quantitative evidence that can shed light on whether residents of rural areas are more concerned about COVID-19 than residents of urban areas.

**Objective:**

This infodemiology study investigated attitudes toward COVID-19 in different Japanese prefectures by aggregating and analyzing Yahoo! JAPAN search queries.

**Methods:**

We measured COVID-19 concerns in each Japanese prefecture by aggregating search counts of COVID-19–related queries of Yahoo! JAPAN users and data related to COVID-19 cases. We then defined two indices—the localized concern index (LCI) and localized concern index by patient percentage (LCIPP)—to quantitatively represent the degree of concern. To investigate the impact of emergency declarations on people's concerns, we divided our study period into three phases according to the timing of the state of emergency in Japan: before, during, and after. In addition, we evaluated the relationship between the LCI and LCIPP in different prefectures by correlating them with prefecture-level indicators of urbanization.

**Results:**

Our results demonstrated that the concerns about COVID-19 in the prefectures changed in accordance with the declaration of the state of emergency. The correlation analyses also indicated that the differentiated types of public concern measured by the LCI and LCIPP reflect the prefectures’ level of urbanization to a certain extent (ie, the LCI appears to be more suitable for quantifying COVID-19 concern in urban areas, while the LCIPP seems to be more appropriate for rural areas).

**Conclusions:**

We quantitatively defined Japanese Yahoo users’ concerns about COVID-19 by using the search counts of COVID-19–related search queries. Our results also showed that the LCI and LCIPP have external validity.

## Introduction

The COVID-19 pandemic has been threatening global health since the end of December 2019. The outbreak has created critical challenges for public health, research, and medical communities [1]. As of July 12, 2021, COVID-19 has affected 220 countries and territories, with over 187 million confirmed cases, and has claimed over 4 million lives [2]. COVID-19 has also disrupted many lives and caused psychological trauma on a large scale [3,4].

As with any outbreak of an infectious disease, the population's psychological reactions play a critical role in shaping the spread of the disease and the occurrence of emotional distress and social disorder during and after the outbreak [5]. Recently, Ahorsu et al [6] developed the Fear of COVID-19 Scale (FCV-19S) by conducting qualitative interviews to assess individuals' fear of COVID-19. Gao et al [7] found that greater concern about COVID-19 (frequent exposure to COVID-19–related social media) was positively associated with adverse mental health outcomes. Furthermore, Su et al’s [8] Twitter-based analysis revealed that spatial-temporal and socioeconomic disparities shaped US residents’ response to COVID-19.

Infodemiology is the science of distribution and determinants of information in an electronic medium, specifically the internet, or in a population, with the ultimate aim to inform public health and public policy [9]. The underlying objective of this field is to measure the pulse of public opinion, attention, behavior, knowledge, and attitudes by tracking what people do and write on the internet [10], such as by analyzing queries from internet search engines to predict disease outbreaks. Bernardo et al [11] published a scoping review in which they assessed the current state of knowledge regarding the use of search queries and social media for disease surveillance, showing their usability. Daughton et al [12] conducted an infodemiology study using social media data from Twitter to identify human behaviors associated with COVID-19 transmission and the perceived impact of COVID-19 on individuals. Mavragani et al [13] provided a methodological framework for using Google Trends in infodemiology by analyzing the value and validity of using Google Trends data.

Worryingly, use of the internet and social media has increased dramatically due to the enforcement of social distancing and stay-at-home orders in many areas, which has led to more search queries for updates on the local COVID-19 situation [14]. However, there is a lack of quantitative evidence about the relationship between these searches for updates and the psychological reactions of the population toward COVID-19. Additionally, one may think that residents of urban areas would show more concern about COVID-19 due to larger crowds and easier access to public transportation. However, concern about COVID-19 is also prevalent in rural areas, given that people who live in rural areas may be more vulnerable to COVID-19 than residents of urban areas [15,16].

Therefore, we aimed to quantitatively reflect the different types of concern in rural and urban areas by analyzing the Japanese public's psychological reactions toward COVID-19 using an infodemiology approach (ie, concern about COVID-19 in search queries). We used Yahoo! JAPAN to get the search queries in this study because it is the largest portal site in Japan, with a significant user base; there are about 70 million and 20 million monthly active users for smartphone and PC, respectively. This covers about 85% of smartphone users and 61% of PC users in Japan. The proportion of female and male smartphone users is 52% and 48%, respectively. About 32% of users are aged 20-39 years, 40% of users are aged 40-59 years, and 28% of users are aged ≥60 years [17]. We first developed two concern indices based on search queries to measure generalized COVID-19 concerns, which we called the localized concern index (LCI) and the localized concern index by patient percentage (LCIPP). We then used these indices to investigate COVID-19 concerns in relation to prefecture urbanization. To evaluate the feasibility of these concern indices, we examined the prefecture-level correlations with several indicators of ruralization and public health outcomes. The Methods section details the process of defining the LCI and LCIPP equations.

## Methods

### Target Queries

First, we explored people's COVID-19 concerns by analyzing search queries over different time periods. We initially established a baseline of common search queries prior to and during the pandemic by selecting the search queries of Yahoo! JAPAN's users from April to May 2019 and from April to May 2020 (we chose to examine queries during April and May due to the Japanese government’s declaration of a state of emergency in April 2020 [18]). Additionally, since older adults (those aged >65 years) are at a significantly greater risk of adverse COVID-19 outcomes [19], we speculated that there might be more COVID-19–related search queries from this group. Therefore, we started the analysis by targeting the search queries of people over 65 years of age. We extracted 100,000 search queries of this older population for the two aforementioned time periods and ranked them in reverse order according to the search counts. A change in time period results in a change in search query ranking; thus, we quantified the difference in ranking by defining the following rank change index:


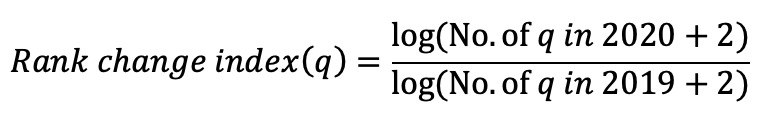


The larger the index, the fewer the search counts for this query in 2019 or the greater the number of search counts in 2020, and vice versa. A constant of two was added to both the numerator and denominator, to avoid instances of zeros (uncountable) in the search counts. Table 1 shows the top five rapidly increasing search queries in April 2020 compared to April 2019. As expected, these terms appeared to be COVID-19–related.

Consistently, of the top 100 queries in the ascending query list, 76 queries contained COVID-19–related keywords (eg, コロナ [corona], マスク [mask]); of these, 33 queries contained prefecture names combined with コロナ感染者 (corona cases). A query pattern, such as the prefecture name plus “coronavirus cases,” clearly displays the prefecture’s information and it also reflects the user's concern about COVID-19 to some extent. Therefore, we chose this query pattern as our target query and sorted the queries by region according to the prefecture names mentioned in the query. Table 2 lists some query samples used in this study.

**Table 1:** Top five rapidly ascending search queries and their rank change index in April 2020 compared to April 2019. The first one, “novel_coronavirus,” is the original data we retrieved from the database.

Search query	Rank change index
novel_coronavirus	18.87
シャープ マスク (Sharp's face mask)	18.74
新型コロナウイルス (novel coronavirus)	17.99
コロナ 感染者数 (corona cases)	17.96
東京都 コロナウイルス感染者 (Tokyo coronavirus cases)	17.63

**Table 2:** Samples of target queries.

Search query	Rank change index
東京都 コロナウイルス感染者 (Tokyo coronavirus cases)	17.63
神奈川県 コロナ感染者 (Kanagawa corona cases)	16.26
埼玉県 コロナウイルス感染者 (Saitama coronavirus cases)	16.18
福岡県 コロナウイルス感染者 (Fukuoka coronavirus cases)	16.11
茨城県 コロナウイルス感染者 (Ibaraki coronavirus cases)	15.84

### Baseline Queries

**Figure 1:**
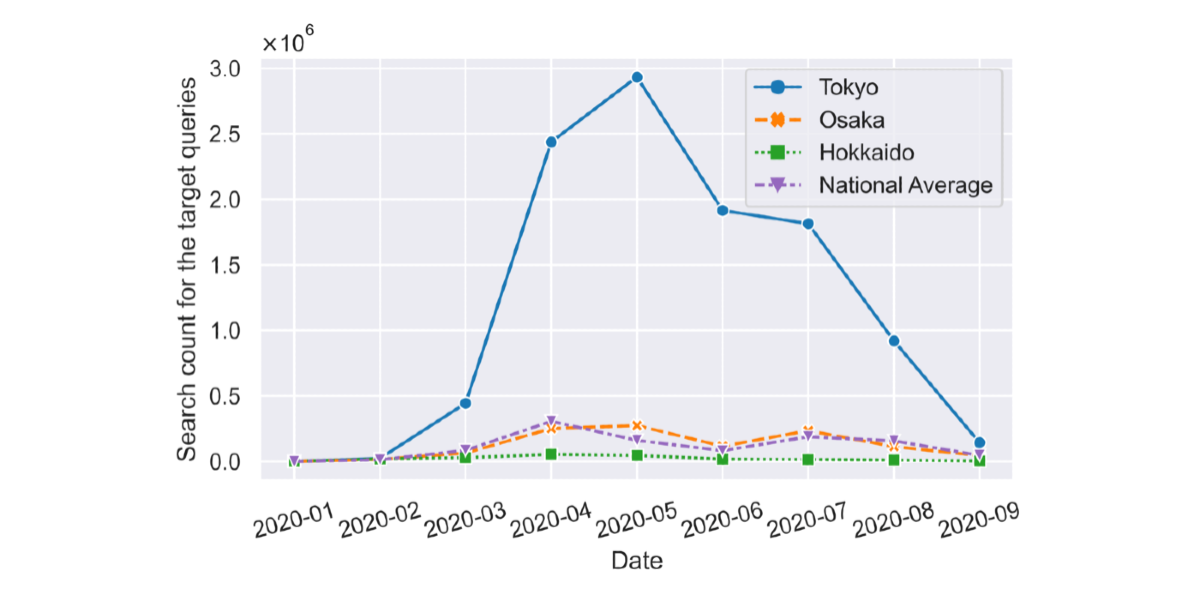
Search counts of targeted queries in Tokyo, Osaka, and Hokkaido, as well as the national average from January to September 2020.

However, when we calculated the search counts of target queries that comprised the prefecture name and “coronavirus cases” from January to September 2020, we found that the search counts in Tokyo were much higher than in the other prefectures, as shown in Figure 1. It is possible that Tokyo’s larger population meant that the frequencies of the general search counts were higher than in less populated prefectures. We speculated that this excessive disparity would inevitably have an impact on our subsequent calculations. To mitigate this effect, we introduced baseline queries for each prefecture that were frequently searched for in that prefecture and that had a relatively stable search count for a certain period. The baseline queries contained the prefecture name and “X,” where “X” referred to any keywords as long as the sample variance of their monthly search counts was as small as possible. For example, for Tokyo, 東京 23区 (Tokyo 23 wards) and 東京 天気 過去 (past weather in Tokyo) were some of the baseline queries, and their sample variances of search counts in these nine months were 499.6 and 683, respectively. In summary, we compared the search counts of possible queries with the above pattern from January to September 2020, and then identified the top three queries with the smallest sample variance during those nine months to form the baseline queries. This was done to balance the impact of excessive disparities by using the quotient of the baseline query and our target query, as the search counts of the target queries were high for urbanized prefectures (such as Tokyo), where the search count frequencies of the baseline queries were also high.

### COVID-19 Concern Indices

Next, our query-based equation to quantify the level of concern about COVID-19—the LCI equation—was defined for each prefecture *pref* as follows:


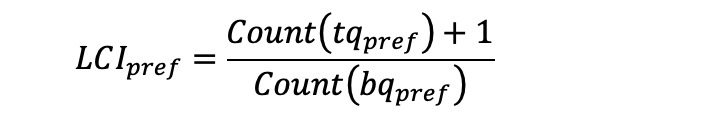


where *tq_pref_* and *bq_pref_* are the target query and the baseline query, respectively, and *Count*(.) is a function that counts the occurrences of a query. We used the logarithmic result for the LCI calculation, which suggests that a higher LCI means a higher frequency of baseline-controlled, prefecture-specified COVID-19–related queries, which in turn reflects a greater level of concern about COVID-19 in that prefecture.

However, the LCI appeared to be greatly influenced by the severity of the COVID-19 situation in each prefecture. For example, urbanized areas, such as Tokyo and Osaka, which are the first and second most populous cities in Japan and the hardest hit, naturally had the highest search counts for target queries and a higher LCI. The COVID-19 pandemic has raised people's anxiety levels [20], and the LCI appears to reflect this attitude in hard-hit, largely urban areas. However, heightened concern about COVID-19 in rural areas [21] does not seem to be reflected in the LCI. Rather, given the lower number of cases in rural areas, it is likely that concern about COVID-19 in rural areas may be related to socioecological variables unrelated to the risk of infection.

For this reason, we attempted to improve our LCI equation to be able to examine COVID-19 concerns beyond the direct influence of actual COVID-19 cases. We argue that this would quantify the prevalence of concern about COVID-19 beyond the risk of actual infection. To do so, we modified the LCI to account for the number of new cases per month in each prefecture by the population of that prefecture to calculate the percentage of infected patients; that is, *Number of monthly new cases/population.*

In summary, the LCIPP for each prefecture *pref* was calculated as follows:


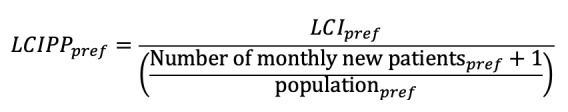


Similarly, we used the logarithmic result for the LCIPP calculation.

### Correlation Indicators

To examine whether the differentiated patterns of associations for the LCI and LCIPP reflected the COVID-19 concerns in rural and urban areas, we examined prefecture-level correlations with both indices. Accordingly, we included the corresponding measures and examined the following aspects, with the corresponding indicators in parentheses: (1) the prevalence of farming (number of farming households, percentage of farmland, rice production), (2) population change (rate of population change), (3) ease of accessibility (reachable areas within one hour from major stations of each prefecture, travel time from Tokyo to major stations in each prefecture), and (4) public health outcomes (reported symptoms, daily outpatients, COVID-19 cases per million residents).

We argue that rural areas can be identified by the higher prevalence of farming households, more farming area, and higher rice production. Conversely, urban areas can be identified by their higher population density, ease of accessibility (the proportion of reachable areas within one hour from major stations of each prefecture), and rate of population change (urban areas should exhibit population growth, while rural areas should show a population decline). Finally, prefectural public health was measured as the number of cumulative COVID-19 cases per 1 million residents (as of September 30, 2020), the proportion of the population that reported general symptoms (non–COVID-19–related), the number of ambulance dispatches, and the number of daily outpatients in 2019. Public health outcomes are able to reflect the current state of local health care to some extent. Since COVID-19 outbreaks would take over existing health care resources, we aimed to find the relationship between public concern about COVID-19 and the current state of local health care through the public health outcomes indicators.

## Results

### LCI and LCIPP Results by Prefecture

**Figure 2:**
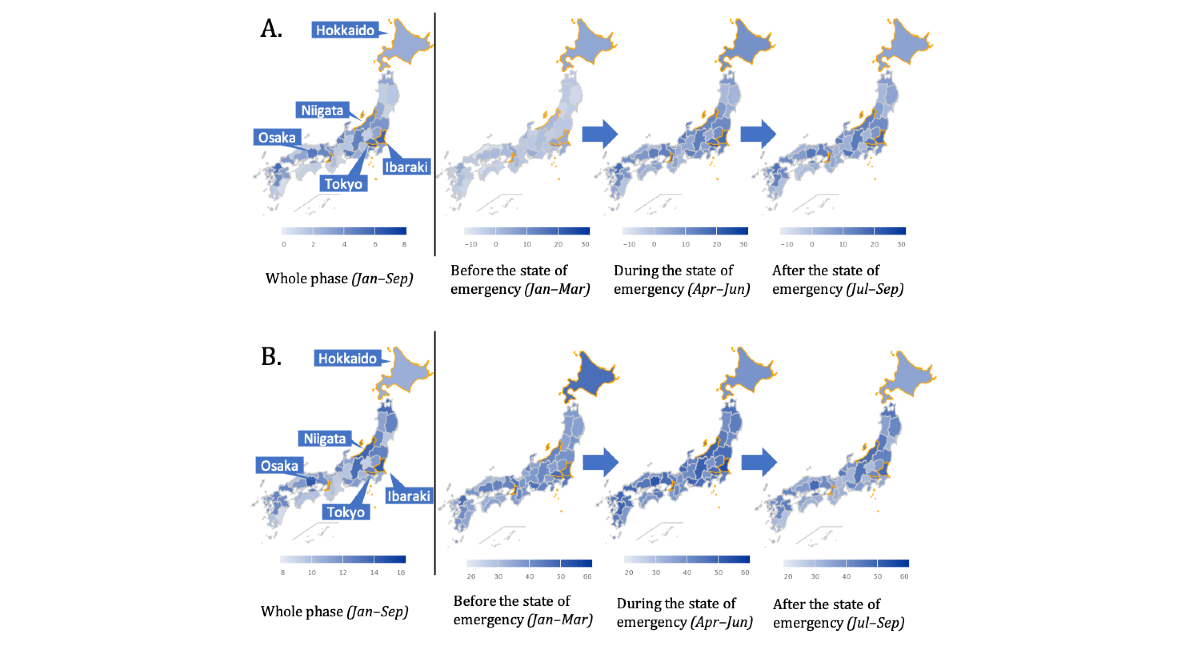
Geographical results. The prefectures with orange boundary lines are the ones mentioned in this study: Hokkaido, Niigata, Ibaraki, Tokyo, and Osaka. (A) Localized concern index (LCI) results. (B) Localized concern index by patient percentage (LCCIP) results.

Figure 2A and Figure 2B show the geographical results of the LCI and LCIPP from January to September 2020, respectively. Table 3 shows the top prefectures with the highest and lowest LCI and LCIPP. These are also shown as the darkest and lightest regions in Figure 2, respectively. In addition, we examined the LCI and LCIPP across three phases following the timing of the state of emergency: (1) before the state of emergency (January-March), (2) during the state of emergency (April-June), and (3) after the state of emergency (July-September). For a breakdown of the LCI and LCIPP by prefecture, please refer to our online supplementary material [22]. The LCI results show that before the declaration of the state of emergency, there was a low level of concern about COVID-19 nationwide. However, with the state of emergency declaration, some prefectures (including Tokyo and Ibaraki) began to show a high level of concern. At the end of the state of emergency, the overall level of concern in the country did not change much, though prefectures such as Hokkaido showed a slight decrease. The LCIPP results show a high level of concern for COVID-19 in Hokkaido even before the national state of emergency was declared. This is because Hokkaido activated COVID-19 response measures at an earlier stage (March 2020) [23], which resulted in a high level of concern for COVID-19 among local residents. With the declaration of a nationwide state of emergency in April 2020, most prefectures also showed a high level of concern, and with the end of the state of emergency, the level of concern declined accordingly.

**Table 3:** Prefectures with the highest and lowest localized concern index and localized concern index by patient percentage.

Search query	Rank change index
東京都 コロナウイルス感染者 (Tokyo coronavirus cases)	17.63
神奈川県 コロナ感染者 (Kanagawa corona cases)	16.26
埼玉県 コロナウイルス感染者 (Saitama coronavirus cases)	16.18
福岡県 コロナウイルス感染者 (Fukuoka coronavirus cases)	16.11
茨城県 コロナウイルス感染者 (Ibaraki coronavirus cases)	15.84

**Figure 3:**
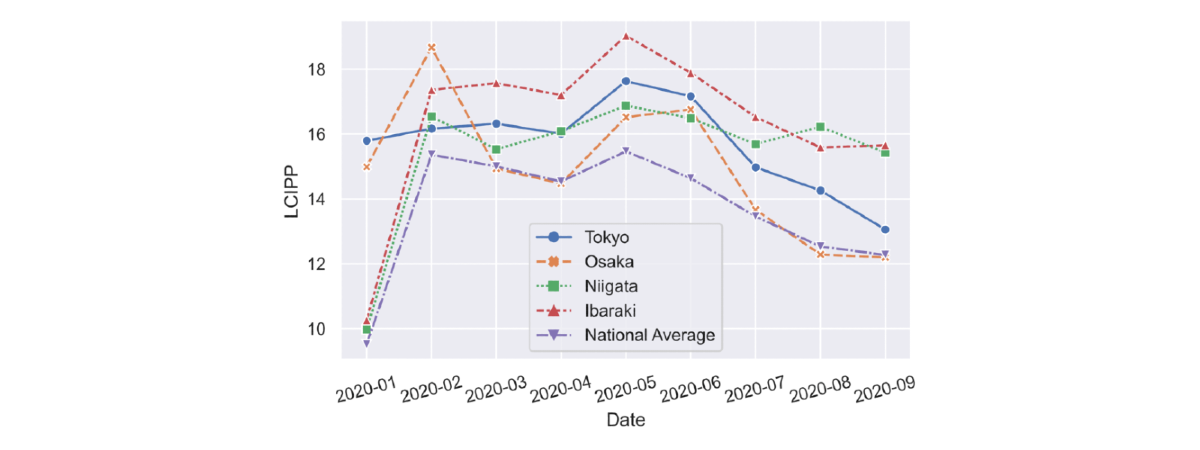
LCIPP results of Tokyo, Osaka, Niigata, and Ibaraki, as well as the national average from January to September 2020. LCIPP: localized concern index by patient percentage.

Of all the prefectures, we selected four to be displayed in Figure 3: Tokyo, Osaka, Niigata, and Ibaraki. We used the number of farming households as a criterion for distinguishing between urban and rural areas. According to a survey by the Statistics Bureau of Japan [24], Tokyo and Osaka have the lowest numbers of farming households in Japan, while Ibaraki and Niigata have the highest numbers. This suggests that Niigata and Ibaraki have relatively lower urbanization rates than Tokyo and Osaka. Nevertheless, Figure 3 indicates that some urban areas (ie, Tokyo and Osaka) have a relatively lower LCIPP than some rural areas (ie, Ibaraki) in terms of the general trends.

### Correlations with the LCI and LCIPP

By calculating the correlation coefficients of LCI and LCIPP on the aforementioned indicators, our results showed that the LCI was significantly correlated with the COVID-19 infection risk (cumulative cases) and urbanized prefectures. The latter was identified through population density and ease of accessibility. The LCI was higher in prefectures that have a high proportion of reachable areas within one hour from their major stations and in prefectures that had growing populations. In contrast, the LCIPP was higher in prefectures that were more rural, as determined by their higher number of farming households and rice production. Table 4 displays the correlation results.

**Table 4:** Correlation coefficients of the whole phase localized concern index and localized concern index by patient percentage and some indicators.

Index and prefecture			Value
**Highest localized concern index**			
	Tokyo	7.04	
	Ibaraki	5.90	
	Fukuoka	5.54	
	Saitama	5.31	
	Okayama	4.92	
**Lowest localized concern index**			
	Miyazaki	0.63	
	Ehime	0.87	
	Mie	0.96	
	Miyagi	1.02	
	Wakayama	1.07	
**Highest localized concern index by patient percentage**			
	Okayama	14.36	
	Ibaraki	14.31	
	Niigata	13.78	
	Nagano	13.48	
	Aomori	13.46	
**Lowest localized concern index by patient percentage**			
	Miyazaki	8.68	
	Ehime	9.18	
	Gunma	9.23	
	Okinawa	9.30	
	Shiga	9.43	

### Age-Based LCIPP Results

**Figure 4:**
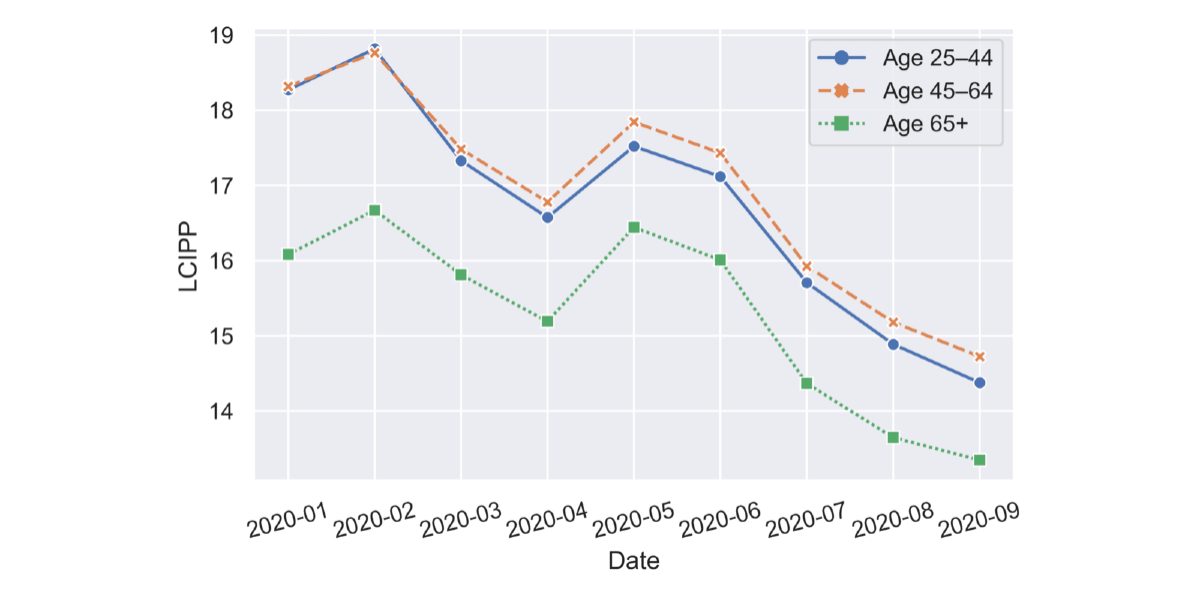
LCIPP results for those aged 25-44 years, 45-64 years, and over 65 years. LCIPP: localized concern index by patient percentage.

We also investigated the LCIPP results for three different age groups: (1) age 25-44 years, (2) age 45-64 years, and (3) age >65 years. At this point, *population* in the LCIPP equation was replaced by the number of people in each age group. Figure 4 shows that older people over the age of 65 years had fewer concerns than those aged 25-44 years and 45-64 years, while those aged 25-44 years and 45-64 years had almost the same level of concern about COVID-19.

## Discussion

### Principal Findings

#### Nature of the LCI and LCIPP

As expected, the LCI appeared to represent the overall concern about COVID-19. To a large extent, this was influenced by the actual prevalence of COVID-19 within the prefecture. Similarly, concern about COVID-19 was heightened in prefectures that are dense, highly accessible, and growing in population. Most likely, these are reflections of urbanized prefectures that have a highly developed infrastructure and that attract the migration of younger workers due to a higher number of job opportunities. As such, these fast-paced, young, and dense prefectures naturally present a greater risk of COVID-19 infection, and the increased concern shown in the search queries that was observed in the LCI is not surprising.

However, once we removed the variance explained by the inclusion of daily increases in COVID-19 cases from the equation, we found the opposite effect. The LCIPP ceased to reflect the COVID-19 risk in urban areas, and there was no significant relationship with the cumulative COVID-19 cases. Furthermore, the pattern of significant correlations revealed an association with the rural prefectures. The larger the proportion of farming households and the greater the rice production, the higher the public concern about COVID-19, which is beyond that explained by the risk of infection. Why does the LCIPP reflect increased ruralization? We posit an explanation in the form of collectivism afforded by farming, specifically in rice-farming societies [25,26]. These societies tend to be more collectivistic and residents have a greater psychological desire to protect the community from internal and external threats. COVID-19 is one such threat; thus, rural communities may have greater vigilance and concern about preventing COVID-19 from becoming prevalent in their community. This is also consistent with prior studies that have established links between COVID-19 concern and collectivism [27] and collectivism with COVID-19 prevention behavior [28].

This explanation has potential implications for policy. Specifically, the discrepancy between the LCI and LCIPP shows that more care must be taken when comparing public attitudes toward COVID-19 between rural and urban communities. If the LCIPP is indeed a result of collectivism and greater vigilance against COVID-19, this may also indicate a broader adoption of preventive measures, such as handwashing and mask wearing. In contrast, the LCI does not seem to be similarly indicative of such concerns. For greater effectiveness, public campaigns that promote such behaviors should therefore use different strategies when targeting rural and urban communities.

Interestingly, we note that the public health measures in Table 3 did not correlate significantly with the LCIPP. This suggests that the preexisting or general health of a prefecture's population does not appear to affect public concern about COVID-19.

#### Preliminary Analysis of the Age-Based Results

Japan, which is one of the fastest aging countries in the world, has the highest proportion of older people worldwide [29]. Emerging studies suggested that older people are more susceptible to COVID-19 and likely to have poor outcomes [19,30,31]. However, we found that individuals aged over 65 years had reduced LCIPP scores, which suggests that their concern might be lower than that of those aged 25-44 years and 45-64 years. Alternatively, this could be a consequence of internet literacy, as users above 65 years of age may have less proficiency in using the internet, or are simply less accustomed to using search terms and queries for topics of concern. However, more research is needed to contextualize this result.

#### Effectiveness of Search Queries as Public Concern Indicators

Finally, we evaluated the usefulness of our method by extracting search queries and combining them with actual COVID-19 infection rates to quantify public concern. Our results showed the correlations for the LCI and LCIPP demonstrate external validity, as they were both associated with constructs that could be explained by previous research. This study joins a growing body of literature that uses web-based search queries to track public health (eg, Murayama et al [32]), and the LCIPP adds the dimension of using prefecture-level infection rates to control for expected outcomes. Therefore, this study was able to effectively quantify public concern at a deeper level, which we propose is explained by the collectivistic psychological tendencies of a society.

### Limitations

We note the limitations of our approach. Our method of extracting prefecture information from search queries relied on searches for the prefecture name + “coronavirus cases,” and not location-based information, such as IP addresses. This may not necessarily represent queries that are only from residents of these prefectures; it may also reflect queries from nonresidents who are interested in the COVID-19 situation in these prefectures (eg, a user who may have family in these prefectures). In addition, although Yahoo! JAPAN has a large user base in Japan, in the broader search engine market, Google still has a significant share. Search queries and results might vary between search engines due to user preferences and service provider settings. Therefore, from the perspective of data diversity, it would be a better choice to consider a combination of search queries from multiple search engines.

### Conclusions

In summary, this study used search queries from Yahoo! JAPAN users to quantify the degree of concern about COVID-19 in rural and urban areas. We first established that Yahoo search queries could be used to quantify COVID-19–related concerns. We then defined the LCI and LCIPP as quantitative indicators of prefecture-level COVID-19 concern. The LCI was indicative of COVID-19 concern in urban areas, whereas the LCIPP appeared to be indicative of COVID-19 concern in rural prefectures. By investigating the relationships between these concern indices and prefecture-level information, we showed that the LCI and LCIPP have external validity. These results suggest that one potential application could be conducting differentiated public campaigns about COVID-19 prevention and misinformation. In light of the different sources of concern in rural and urban areas, such campaigns could adopt different risk communication strategies in these areas.

## Abbreviations

LCI: localized concern index
LCIPP: localized concern index by patient percentage
